# Understanding the Reproductive Biology of *Angelonia integerrima* Spreng. (Plantaginaceae), a Native Southern Brazilian Plant with Horticulturally Desirable Traits

**DOI:** 10.3390/plants14050663

**Published:** 2025-02-21

**Authors:** Júlia de Moraes Brandalise, Fernando H. Calderon-Quispe, Rafael Becker, Renan Pittella, Jessica Pinto Moura, Rosana Farias-Singer, Rodrigo Bustos Singer

**Affiliations:** 1 Graduate Program in Botany (PPGBOT-UFRGS), Universidade Federal do Rio Grande do Sul, Porto Alegre 90035-003, RS, Brazil; fcalderonq@unsa.edu.pe (F.H.C.-Q.); beckerr92@gmail.com (R.B.); renanpittella@gmail.com (R.P.); jessicamoura968@gmail.com (J.P.M.); rbsinger1@yahoo.com (R.B.S.); 2 Porto Alegre Botanical Garden, Secretaria do Meio Ambiente e Infraestrutura do Estado do Rio Grande do Sul, Porto Alegre 90119-900, RS, Brazil; rosanafarias@gmail.com

**Keywords:** biodiversity, Brazil, gardening, herkogamy, native bees, Pampa biome

## Abstract

*Angelonia* Bonpl. (Plantaginaceae) is a neotropical genus of ornamental interest, with some Mexican species already commercialized in the horticultural trade. *Angelonia integerrima* Spreng. is the only species of the genus native to Southern Brazil, and, despite its high ornamental potential, its reproductive requirements are unknown. Flower features and natural pollination were studied in the field in three localities within Southern Brazil. Pollination was recorded through pictures and videos. The breeding system was studied through controlled pollinations applied to plants excluded from pollinators. Germination was studied under controlled temperatures (20°, 25°, and 30 °C). According to our results, flowers are protandrous and keep their fresh appearance for up to nine days. The flowers produce oils in a pair of trichromes located inside the corolla. The plants are pollinator-dependent and self-compatible. The sole pollinators are oil-gathering solitary bees, *Centris trigonoides Lepeletier*, *1841* and *Centris* sp. (Apidae), that pollinate the flowers while collecting the floral oils. Germination proceeds better at 25 °C, reaching success of 50%. The domestication of this plant for horticultural purposes in Southern Brazil would be desirable not only for its ornamental characters but also for ecosystemic reasons since the species is already adapted to environmental conditions and its flowers offer resources for native, solitary bees.

## 1. Introduction

**Figure 1:**
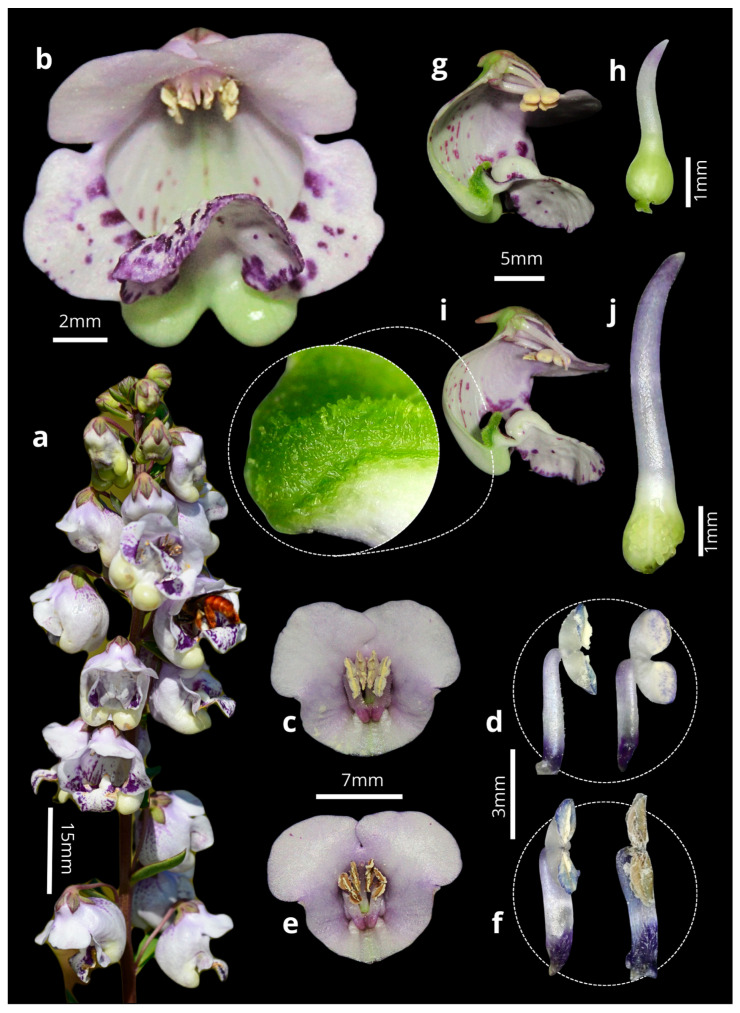
(**a**) Inflorescence of *Angelonia integerrima* Spreng. (**b**) Frontal view of the flower. (**c**) Longitudinal section of the flower in the first phase of anthesis. (**d**) Anthers at the beginning of dehiscence. (**e**) Longitudinal section of the flower in the second anthesis phase. (**f**) Anthers at the end of dehiscence. (**g**) Cross-section of the flower in the first phase of anthesis. (**h**) Short style. (**i**) Cross-section of the flower in the second phase. (**j**) Long style and stigma.

*Angelonia* Bonpl. (Plantaginaceae) is a genus with significant ornamental potential. Mostly associated with savannas and grassland vegetation. Approximately 30 described species are distributed from Mexico to the southern region of Brazil [1]. In Brazil, there are 20 species, with the highest diversity concentrated in the Cerrado (15 species) and Caatinga (11 species) biomes [2]. Despite this, commercially available species in Brazil are native to Mexico (especially *Angelonia angustifolia* Benth.). In the grassland vegetation in the southern part of Rio Grande do Sul, there is only one species: *Angelonia integerrima* Spreng. (commonly known as “*violeta-do-campo*”). This species is found throughout the Pampa Biome in rocky outcrops and stony fields. It is a herbaceous perennial plant with a wine-colored stem and subsessile, linear, opposite leaves. The flowers range from white to lilac with purple spots, arranged in terminal racemes, blooming between October and March (Figure 1a) [3]. This group of plants normally produces floral oils, an essential lipid resource for native solitary oil-collecting bees, which use it to nourish their larvae and line their nests [4]. So far, the pollination and breeding system of five *Angelonia* spp. (*Angelonia bissacata* Benth., *Angelonia cornigera* Hook F., *Angelonia hirta* Cham., *Angelonia hookeriana* Gardn ex Benth., and *Angelonia pubescens* Benth.; all from Northeastern Brazil, within the Caatinga Biome) have been studied in detail [5,6]. These species present trichomatic elaiophores (oil-secreting glands) and—in agreement—are exclusively pollinated by oil-gathering, solitary bees of the genus *Centris* (Apidae) [5,6]. Indeed, all species studied so far present protandrous flowers that act first as pollen donors and later as pollen receivers and (except for *A. pubescens*) are self-incompatible; that is, the flowers of most species fail to set fruit if pollinated with pollen from the same individual [5,6].

The ornamental potential of a plant species is attributed to several reasons, including its morphological characteristics, ease of adaptation to various planting conditions such as garden beds and pots, and simplicity in fertilization [3,7]. In ornamental horticulture, species are also selected for rapid and vigorous germination, accelerated growth, and high reproductive output [8]. Artificial selection favors species germinating more quickly and in greater quantities [9]. Some exotic species introduced through horticulture may become invasive, threatening native flora [10]. In this context, the use of native species in South American ornamental horticulture, such as *A. integerrima*, emerges as a sustainable and promising alternative, replacing exotic species that could potentially become invasive [11]. Urban gardens account for 20–30% of urban areas and serve as refuges for habitat and resources in an increasingly resource-scarce environment. The cultivation of native plant species in these gardens has proven effective in supporting a more diverse and abundant native pollinator fauna [12]. To achieve this goal, it is essential to understand the reproductive biology of the potential target plant species and the most effective and economical propagation methods. Thus, using *A. integerrima* (and any other native plant species) as an ornamental plant in southern Brazil could also provide an ecological benefit by supplying essential floral resources for native pollinators. However, native species like this remain underexplored, with limited knowledge of their germination features, reproductive systems, and pollination.

This study will explore these characteristics of *A. integerrima*, justifying and highlighting the potential of this species for the ornamental plant market. The questions preceding this study are as follows: (1) What is the floral lifespan, and do flowers present any strategy promoting cross-pollination? (2) What is the reproductive system of the species? (3) Which animal species perform the pollination? (4) Does the study species depend on these pollinators for fruiting? (5) What is the optimal temperature for germination? (6) Do different populations exhibit distinct germination patterns? (7) Can *A. integerrima* be effectively propagated through germination for cultivation purposes? By addressing these questions, it will be possible to understand the reproductive biology of the species, properly evaluate its potential for horticulture, and develop propagation protocols. Based on the preceding literature dealing with other Brazilian species [5,6], we hypothesize that (1) flowers may be protandrous and present a long lifespan [5,6]. (2) The species is self-incompatible, as is the case for most species already studied [5,6]. (3) The main pollinators are oil-collecting bees, and (4) the species depends on these agents for fruiting. (5) There is an optimal temperature that maximizes both the quantity and speed of germination, and this temperature is likely consistent across all populations of the species. (6) Germination effectiveness may be influenced by population context, with larger populations in more conserved habitats exhibiting greater vigor compared to those in anthropogenic environments. (7) Germination is an economical, rapid, and efficient method of propagation, especially when compared to in vitro techniques, which have also been proposed for this species [13].

## 2. Results

### 2.1. Anthesis and Dichogamy

**Figure 2:**
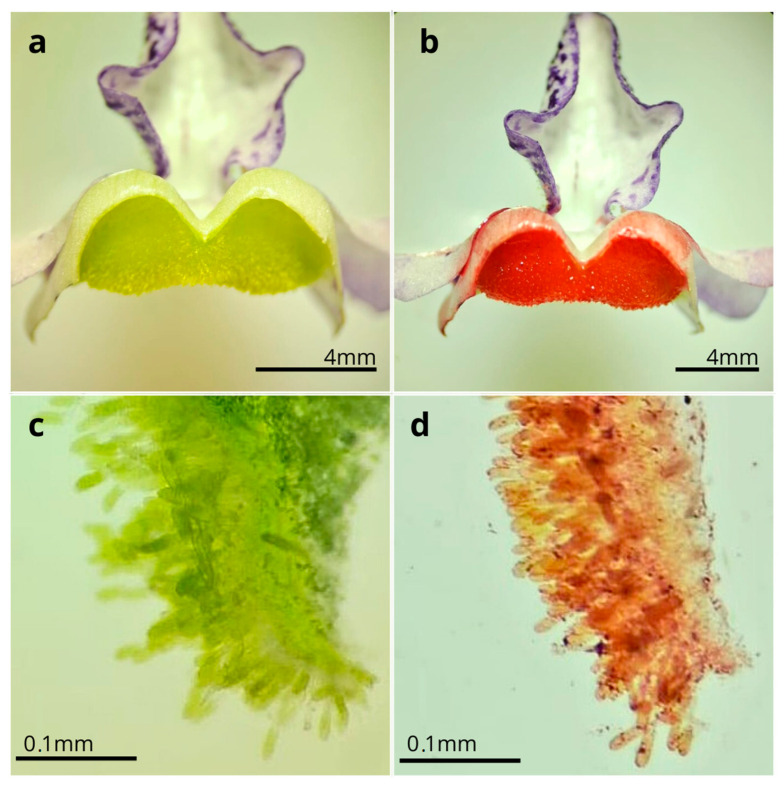
Elaiophores of Angelonia integerrima. (**a**) Cross section of fresh trichomatic elaiophores, without Sudan IV. (**b**) Reaction of oils with Sudan IV. (**c**) Anatomical section of trichromatic elaiophores. (**d**) Anatomical section of lipids from elaiophores stained with Sudan IV.

The flowers of *A. integerrima* last up to nine days ($\overset{-}{x}$ = 8 ± 0.85; n = 10); they are zygomorphic ($\overset{-}{x}$ = 1.56 ± 0.24 cm long; n = 10), with a coloration ranging from white to purple, featuring wine-colored spots on the lip and two oil sacs located in the posterior region. Each flower has two pairs of didynamous stamens, and the style is central and crowned by a simple stigmatic surface (Figure 1b). The inflorescences are terminal, of the raceme type ($\overset{-}{x}$ = 13.83 ± 4.08 cm long; n = 10), and contain many flowers ($\overset{-}{x}$ = 41.4 ± 16.78 flowers; n = 20), which bloom gradually from the base to the apex of the inflorescence. The species exhibits dicogamy (specifically, protandry), where the anthers dehisce before the stigma becomes receptive (Figure 1c–j). Anthesis is thus divided into two phases: (1) On the first day of anthesis, the anthers undergo longitudinal dehiscence ($\overset{-}{x}$ = 1.54 ± 0.49; n = 10), with the outermost pair of anthers dehiscing first, followed by the second pair, thus exposing the whitish pollen (Figure 1c–f). (2) Starting on the fourth day ($\overset{-}{x}$ = 4.1 ± 0.3; n = 10), the style elongates, becoming more prominent than the anthers, and the stigma becomes receptive until the end of the anthesis, by which time the anthers no longer contain pollen (Figure 1g–j). The Sudan IV reagent test confirmed the presence of lipids/floral oils located in the cushion-like elaiophores. Thus, the results indicate that the elaiophores in the study species are of the trichomatic type (Figure 2).

### 2.2. Pollination

**Figure 3:**
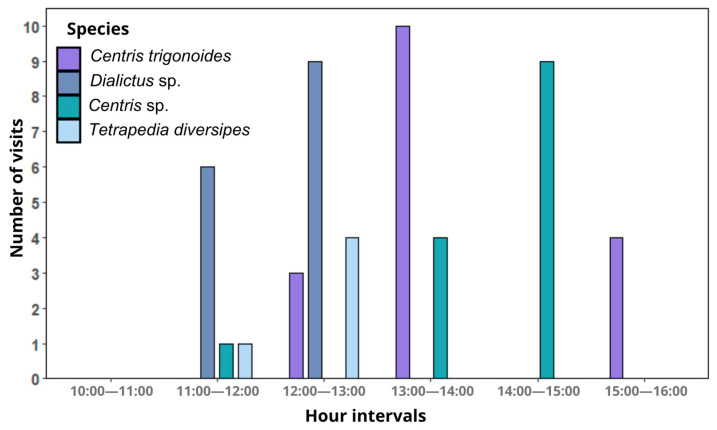
Frequency of visits of bee species to *Angelonia integerrima* flowers at 60 min intervals.

**Figure 4:**
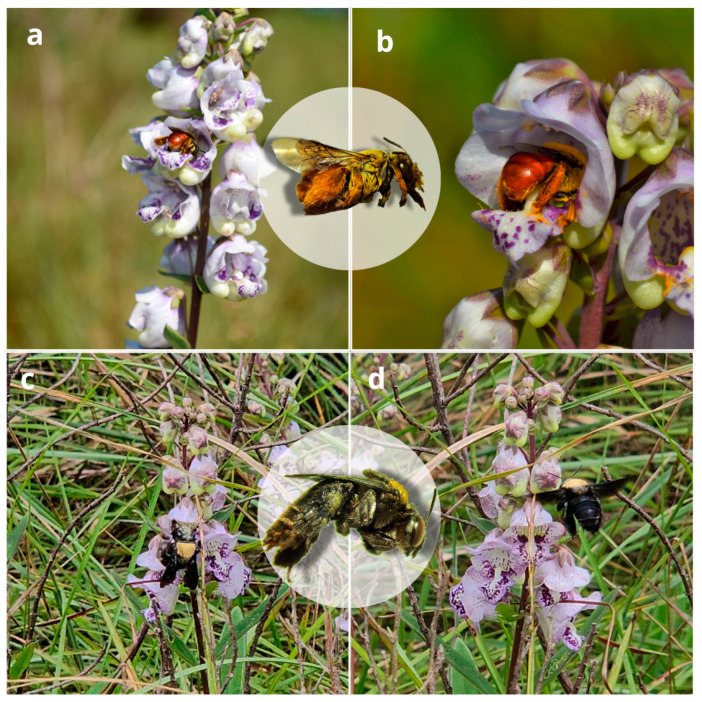
Pollinating bees of *Angelonia integerrima*, (**a**,**b**) *Centris trigonoides Lepeletier*, *1841*(Centridini), and (**c**,**d**) *Centris* sp. (Centridini).

Four bee species were observed interacting with the flowers of A. integerrima: Centris trigonoides (Centridini), Centris sp. (Centridini), Dialictus sp. (Halictini), and Tetrapedia diversipes Klug, 1810 (Tetrapediini). Bee activity was concentrated mainly between 12:00 and 14:00 (Figure 3). Centris trigonoides exhibited the highest number of interactions, totaling 59 visits, which occurred primarily between 13:00 and 14:00, with an average duration of 2.44 s per flower (Table 1, Figure 3). Only females were recorded during these visits. During foraging, the bees land on flowers in search of floral oils stored within the trichomes of the elaiophores in the inner region of the corolla. To collect the oils, they scrape the trichomes with their forelegs, while the posterior dorsal region of their thoraxes (especially the scutum and scutellum) comes into contact with the reproductive floral whorls, allowing pollen to adhere to the bee’s fur (Figure 4). This pollen is subsequently transferred to stigmas when pollen-loaded bees visit flowers in the female phase (Figure 1b). The second species with the highest number of interactions was Centris sp., with 48 visits, occurring mainly between 14:00 and 15:00, with an average duration of 1.75 s per flower (Table 1, Figure 3). The foraging behavior of Centris sp. was similar to that of C. trigonoides. Tetrapedia diversipes visited the flowers 10 times, collecting both oils and pollen (Table 1). In contrast, Dialictus sp. (Halictidae) displayed a distinct behavior, focusing exclusively on pollen collection. This species performed the longest visits, with an average duration of 55.76 s, but were unlikely to visit flowers in the female phase and promote pollination (Table 1). The foraging behavior for each species is detailed in Supplementary Material (Video S1).

### 2.3. Breeding Systems and Reproductive Success

Reproductive system tests showed fruit set only in the self-pollination (geitonogamy) and cross-pollination treatments, while no fruit formation was observed in emasculation and control treatments (Table 2). So, there is no evidence that *A. integerrima* can neither automatically self-pollinate nor produce fruit and seeds through apomyxis. These results indicate that the species is self-compatible but depends on pollinators for fruit development. Furthermore, fruit sets from experimental hand-crosses and natural conditions showed no statistically significant difference in the Kruskal–Wallis test (*p* = 0.7469), suggesting that pollination performed by pollinators reflects the species’ maximum fruiting potential. Seed production did not show a significant difference between the self-pollination (geitonogamy) and cross-pollination treatments, as indicated by the ANOVA (Table 2). This suggests that both insect-promoted autogamous and allogamous pollination modes resulted in similar seed production. Similarly, fruit formation was statistically comparable between the two pollination types (self-pollination and cross-pollination), with an ANOVA *p*-value of 0.287, indicating no significant difference between the pollination types.

### 2.4. Fruit and Seed Features and Germination Tests

**Figure 5:**
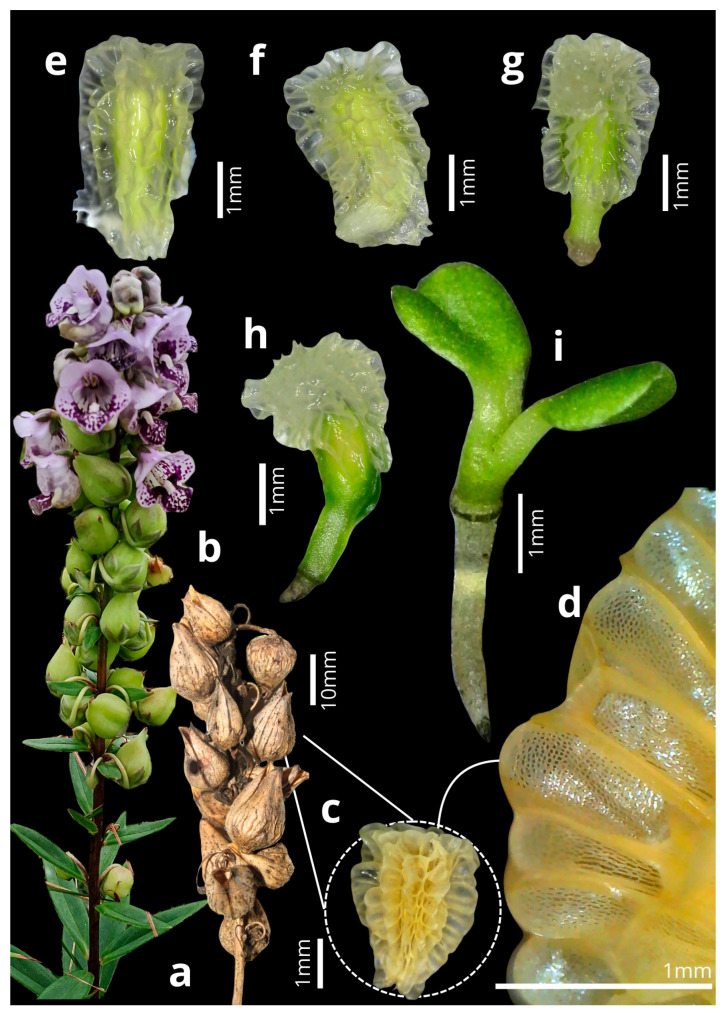
Fruit development and germination of *Angelonia integerrima*. (**a**) Immature fruits. (**b**) Fruit dehiscing. (**c**) Seed. (**d**) Seed detail. (**e**) Seed soaked in water, with the green embryo (day 1). (**f**) Hypocotyl curvature (2–3 days). (**g**) Hypocotyl development (2–3 days). (**h**) Cotyledon development is still enclosed by the seed (4–5 days). (**i**) Seedling completely detached from the seed, with developed cotyledons and hypocotyl (5–7 days).

*Angelonia integerrima* typically presented 6–34 ($\overset{-}{x}$: 17 ± 6.64; n = 20) fruits per raceme (Figure 5b). The ripe fruits are brown, ovoid dehiscent capsules approximately 1.76 cm long and 1.04 cm wide, and open along two longitudinal fissures exposing the seeds (Figure 5b). The seed shape can vary from ovoid to pyramidal-triangular, with a sandy color when dry and translucent when soaked in water (Figure 5c–e). The testa is highly ornamented, with translucent-colored projections evenly distributed across the entire seed coat (Figure 5d). The seeds measure 2.13–3.27 mm long ($\overset{-}{x}$: 2.55 ± 0.28; n = 50) and 1.2–2.18 mm wide ($\overset{-}{x}$: 1.71 ± 0.19; n = 50). The seeds began to germinate between the third and ninth day of the experiment. Germination lasts approximately five to seven days, with the embryo turning green on the first day (Figure 5e). Subsequently, the hypocotyl emerges from the seed and begins development until approximately the third day (Figure 5f,g). On the fourth to fifth day, the cotyledons begin to expand and detach from the seed coat, fully exposing the seedling (Figure 5h,i).

Comparing the locations (Table 3), germinability was higher in MSP samples (MSP-JB *p*-value = 0.008, MSP-MS *p*-value = 0.034), and there were no statistical differences in germinability between JB and MS samples. While the GSI was higher in MSP compared only to JB (*p* = 0.01), the MS differs between the other locations. The synchrony index showed no statistical difference among populations. On the other hand, comparing temperatures (Table 4), at 25 °C germinability was the highest (25–20 °C, *p*-value = 0.004; 30–25 °C, *p*-value = 0.003; c-25 °C, *p*-value = 0.00001); the other temperatures showed no statistical difference in germinability. The GSI was similar, having the highest and statistically different value at 25 °C (25–20 °C, *p*-value = 0.0007; 30–25 °C, *p*-value = 0.0004; c-25 °C, *p*-value = 0.0001), while synchrony tested a difference only between 25 and 30 degrees (*p*-value = 0.01).

## 3. Discussion

### 3.1. Anthesis and Dichogamy

The average length of the inflorescences of *A. integerrima* (13.83 cm), combined with the intense violet color and the striking appearance of the moderately-sized flowers (1.58 cm in diameter), contributes to the high ornamental potential of the species. The flowering period is long, occurring from October to March, and the longevity of the inflorescence is notably prolonged, from the anthesis of the first flower to the senescence of the last. This characteristic not only enhances its ornamental value but also increases the exposure time of the inflorescence, thereby improving the chances of pollination. The floral morphology of Plantaginaceae has long been recognized for its ornamental value. Genera such as *Mecardonia* Ruiz & Pav., *Antirrhinum* L. (snapdragon), and *Digitalis* L. (foxglove) exemplify plants with well-established commercial applications, both ornamental and medicinal [14,15,16]. In particular, *Digitalis purpurea* L. is cultivated worldwide, used as a cut flower, in ornamental beds, and for medicinal purposes [16]. Its inflorescences and flowers, similar to those of *A. integerrima*, are striking, with gradually opening flowers that exhibit a purple color with spots ranging from pink to purple. In agreement with precedent studies on *Angelonia* species from Northeastern Brazil, *A. integerrima* is also protandrous, a feature that, to some degree, promotes cross-pollination [5,6] by temporarily separating the flower’s pollen donor and pollen receiver functions (but see below in Pollination and Breeding System). Remarkably, the reported floral lifespans in Northeastern Brazil *Angelonia* spp. are shorter (up to seven days) than those of *A. integerrima* (up to nine days), and, consequently, in the former species, the male phase is one day longer than the female phase, in contrast with *A. integerrima* [5,6].

### 3.2. Pollination and Breeding System

The interactions between oil-producing plants and oil-collecting bees are particularly abundant in the Neotropics. In Brazil, these plants account for 2–9% of angiosperms, while oil-collecting bees represent approximately 20% of the neotropical bee fauna [4]. Females of these solitary bees use floral oils to line their nests and feed larvae along with pollen, relying on morphological adaptations on their legs or sterna, such as variations in size and the presence of pilosity, to facilitate the collection and storage of these oils [17]. The main pollinators of *Angelonia integerrima* are *Centris* (Apidae: Centridini), which is the most frequent and effective observed pollen vector. We conclude that, at the study site, *C. trigonoides* and *Centris* sp. are the primary pollinators of *A. integerrima* based on the following factors: (a) these species exhibited the highest number of flower interactions, favoring cross-pollination (Table 1); (b) their body positioning during visits was similar, enabling the dorsal region to contact both anthers and stigma while visiting flowers in both phases (Figure 4, Supplementary Material Video S1); (c) both species visited flowers in both stages of anthesis, acting as both pollen donors and receivers. This behavior is observed in the Supplementary Material Video S1, where *C. trigonoides* and *Centris* sp. visit flowers at both the base and apex of the inflorescence, interacting with flowers from both phases. Unlike other species such as *Tetrapedia diversipes* and *Dialictus* sp., which primarily seek flowers in the pollen donation phase and remain on the same flower for extended periods (Supplementary Material Video S1, Table 1). In the case of *Tetrapedia diversipes*, the position adopted for foraging on the flower indicates pollen collection only. In rare instances, the species sought oil but was not effective in pollination due to its very small size (Supplementary Material Video S1). On the other hand, *Dialictus* sp. always visited only pollen-donating flowers and remained on the same flower for extended periods (Supplementary Material Video S1, Table 1); and (d) their body size is ideal for fitting the flower, allowing efficient oil collection while performing pollination (Figure 4). This is in full agreement with preceding studies with *Angelonia* species from the Caatinga Biome, where species of *Centris* bees were the sole effective pollinators [5,6]. Remarkably, *Centris* bees that pollinate *A. integerrima* carry the pollen onto the dorsal part of the thorax (scutum and scutellum) (Figure 4). This is in marked contrast with the pollination process in the *Angelonia* spp. from the Caatinga Biome, where a combination of flower size and shape, as well as pollinator size, promotes that the pollen of these species is spread onto the head of their pollinators instead [11,12]. As a whole, oil-collecting bees are essential for the survival and propagation of *Angelonia* spp. On the other hand, some oil-gathering bees have likely specialized in collecting oils from specific plant species, rendering the latter essential for the survival of the insects [4]. From the plant’s perspective, the intensity of interaction with pollinators depends on the reproductive system and the efficiency of these agents. Notably, plants of the Mexican *A. angustifolia* cultivated in Porto Alegre also attract females of *Centris trigonoides*, supporting the strength of the relationship between *Angelonia* species and oil-gathering bees. It seems plausible that other studies on the pollination of other Brazilian and non-Brazilian species will evidence similar results. Preceding studies have evidenced that *Angelonia salicariifolia* Bonpl., *A. pubescens*, *Angelonia campestris* Nees & Mart., and *A. cornigera* are self-incompatible, thus requiring cross-pollination for fruit production [5,6]. In contrast, *A. integerrima* is self-compatible but pollinator-dependent (Table 2), requiring interaction with pollinators to produce fruits. Self-compatibility coupled with pollinator-dependency was previously recorded for *A. pubescens* [5]. Pollinator behavior achieves the species’ maximum fruiting capacity (*p* = 0.7469), highlighting the high efficiency of this interaction. Furthermore, since the inflorescence of *A. integerrima* holds flowers in both reproductive phases at the same time (pollen donor and receiver phases) and the species is self-compatible, the probability of fruiting and the production of viable seeds is higher in the case of insect-promoted self-pollinations [18], unlike the case in most studied species from Northeastern Brazil [5,6].

### 3.3. Germination Tests

**Figure 6:**
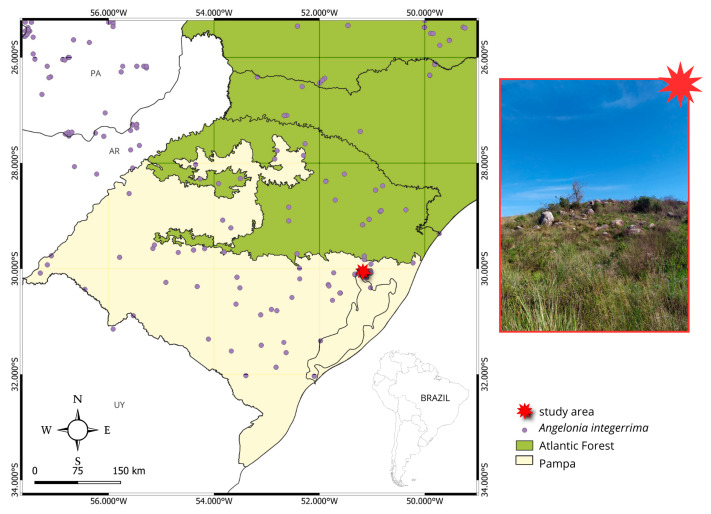
Study area and distribution map of *Angelonia integerrima*.

The seeds of *Angelonia* are classified as crystal-reticulate, characterized by hyaline ridges formed by testa epidermis growth, which provides resistance [19]. In the Plantaginaceae, seed features are important taxonomic tools. *Angelonia* seeds are relatively large (<2 mm) with a hyaline reticulate surface, as seen in *A. integerrima*, one of the largest seeds in the genus (Figure 5d) [20]. Seed ornamentation varies significantly within the genus. For instance, *A. salicariifolia* exhibits longitudinally arranged hyaline cells with small fissures and projections, called microcilia [21]. Conversely, *A. integerrima* seeds are fully covered with small fissures that may enhance air passage and facilitate dispersal, explaining its wide distribution (Figure 5d and Figure 6) [22]. Many Plantaginaceae seeds, including *A. integerrima*, are adapted for anemochory, a dispersal mechanism common in pioneer species of open environments, though less frequent in tropical regions [20,22].

This study is the first to analyze the germination process and optimal temperature for *Angelonia*, with implications for ornamental cultivation. The ideal temperature of 25 °C maximizes germination quantity and speed, with the highest germination speed index (GSI) of 5.19. The optimal range of 20–25 °C is consistent with findings for other Plantaginaceae species, such as *Plantago ovata* Forssk. and *Plantago crassifolia* Forssk [23,24]. Temperature influences germination by affecting enzymatic and metabolic activity, impacting both the speed and the number of seeds germinated [25,26]. Seeds that germinate faster and more synchronously at optimal temperatures, reduce the susceptibility to diseases and increase colonization efficiency [27,28]. Germinability peaks at 25 °C (49.33%), dropping by nearly half at 20 °C and 30 °C, highlighting the species’ temperature tolerance and ecological adaptability [29,30]. In the MSP, a conservation unit with minimal human impact, seeds have shown the highest germinability (41.75%), followed by MS (26.25%), a granite hill with urban and native vegetation [31]. The superior performance of MSP seeds highlights the importance of conservation units in preserving native populations, providing valuable data for breeding programs and genetic resource conservation [32]. Understanding the germination performance of ornamental species is a crucial step preceding domestication and commercialization. To achieve plants with specific quality standards, it is essential to use viable propagation methods. Wild species are valuable germplasm sources, providing genes that can be used to enhance traits in cultivated plants [33]. The observed germination rates are high (49.33%), suggesting that the study species can be successfully propagated from seeds. This approach offers the advantage of increased genetic diversity and reduced production costs. Additionally, *A. integerrima* seeds do not exhibit dormancy, which simplifies the germination process and further lowers costs, as no pre-germination treatments are required. Germination occurs rapidly, typically within the first three days after sowing. Furthermore, the cytological stability of *A. integerrima* supports the selection of future phenotypes for both commercial purposes and conservation efforts [34]. *Angelonia integerrima* is widely distributed across the state of Rio Grande do Sul. This region is characterized by complex and diverse geological formations, with significant variations in soil types, vegetation, and climate [35]. The species is found in both anthropogenic and native areas, including those studied here, demonstrating its adaptability to various conditions. Germination is a cost-effective and rapid propagation method, particularly suitable for species like *A. integerrima* that exhibit high adaptability [29]. In addition to its ease of germination, the seedlings showed high ease of cultivation, with mature plants blooming and fruiting within the first year after germination (personal observation). Given the escalating climate crises and increasing urbanization, the horticultural market trend is shifting towards native species that require low maintenance and exhibit high tolerance to harsh environmental conditions [36]. Thus, the study plant presents a set of features that make it desirable for such purposes.

## 4. Materials and Methods

### 4.1. Study Area

The study was conducted in three areas with natural populations of the species in the municipality of Porto Alegre, Rio Grande do Sul, Brazil: the Jardim Botânico de Porto Alegre (Porto Alegre Botanical Garden, hereafter, JB) (30°03′14.2″ S 51°10′34.2″ W), Morro Santana (hereafter, MS) (30°03′11.9″ S 51°07′19.4″ W), and the Refúgio da Vida Silvestre Morro São Pedro (hereafter, MSP) (30°10′32.5″ S 51°06′22.1″ W) (Figure 6). The three localities, although separated by an average of 12 km, present some differences in structure. The JB is the smallest population studied, in a small fragment of grassland vegetation, with exotic and native species together, which does not undergo frequent management. MS is a granite hill in Porto Alegre nearby an already urbanized region, where anthropogenic fires frequently occur [37]. There is also a mosaic between native vegetation and urban use. At MSP, the plants occur in the Conservation Unit, with a grassland area of 35 ha. Like MS, the MSP also undergoes fire management, but human activity is lower. This region is part of the ecological system of interior sub-montane grasslands, characterized by the dominance of Poaceae and Asteraceae distributed at altitudes between 30 and 150 m [35]. The slopes are moderate, and the soils are deep but with low fertility and rarely exposed [35]. The plant vouchers are deposited in the herbaria ICN (Universidade Federal do Rio Grande do Sul) and HAS (*Herbário Alarich Rudolf Holger Schultz*, SEMAI/RS).

### 4.2. Anthesis and Flower Features

To determine floral longevity and the duration of each reproductive phase, individuals were cultured ex situ. Twenty-five flowers (from five individuals) were isolated from pollinators using tulle bags and monitored daily. Anthesis was considered to begin when the floral bud fully opened and to end when the flower fell. For the observation of dichogamy, the onset and end of anther dehiscence and stigmatic receptivity were recorded daily (n = 25). The beginning of the pollen donation phase was marked by the release of pollen from the thecae and ended when no pollen remained in the anther and the stigma became receptive. To test stigmatic receptivity, drops of hydrogen peroxide were applied to the stigmatic surface on each day of the flower’s anthesis. The stigma was considered receptive when bubble formation occurred [38]. Fresh flowers (n = 10) were collected and immersed in Sudan IV for lipid detection in elaiophores. Anatomical sections were made in the elaiophore region to obtain a more detailed view of the structure [39].

### 4.3. Pollination Observation

Pollinator observations were conducted in the field across the three study areas during the flowering period between October and March of 2022 and 2024. A total of 40 h were spent observing the pollination process and pollinators. Observations were carried out between 10:00 AM and 4:00 PM, during which pollinator activity ceased to be observed. Inflorescences were randomly selected by each observer. All visits were recorded to document the time spent on the flower and inflorescence as well as foraging behavior. Insects were considered pollinators, rather than mere visitors, when they touched both the anthers and stigma of flowers at the different reproductive stages. Insects were collected for subsequent identification and deposited at the Museum of Natural Sciences (MCN), Porto Alegre, Brazil.

### 4.4. Breeding Systems and Reproductive Success

Reproductive system tests were conducted in situ within the JB population. Floral buds were isolated with tulle bags before anthesis to prevent pollinator interaction and pollen contamination. Four treatments were applied to define the reproductive system: (1) manual self-pollination (geitonogamy), flowers in the female phase were pollinated with pollen from another flower, in the male phase, of the same individual; (2) manual cross-pollination, where flowers were pollinated with pollen from another individual; (3) emasculation, with the removal of anthers before pollen release; (4) control, where flowers were bagged until the end of anthesis without any manipulation. Thirty flowers were used for each treatment (totaling 120 flowers, as a whole). A total of 17 individuals were used for these controlled pollinations. A minimum distance of 5 m between individuals was used to avoid the possibility of vegetative reproduction. Approximately one month after the tests were applied, fruits were collected and counted for each treatment. To assess natural reproductive success, 20 inflorescences (from 20 individuals) with mature fruits were analyzed, and fruit set success was calculated as the ratio of the number of flowers per inflorescence to the number of fruits formed under natural conditions.

### 4.5. Germination Tests

The germination tests were conducted at the Seed Bank Laboratory of the Porto Alegre Botanical Garden (*Laboratório Banco de Sementes no Jardim Botânico de Porto Alegre*). The collected seeds were stored at room temperature until the beginning of the experiment in January 2024. Before the germination experiment, the seeds were sanitized with a triple wash of running water and 1% sodium hypochlorite. Afterward, the seeds were placed in a Gerbox container with Germitest paper moistened with 10 mL of deionized water. Each box received 25 seeds, with four replications per locality, totaling 100 seeds per treatment, per locality. Three temperatures were tested: 20 °C, 25 °C, and 30 °C with a 12 h photoperiod in a germinator (Tecnal TE-4020 LED). A control test (mean 29.2 °C) was conducted at room temperature and natural photoperiod, with the same number of replications [29]. The seeds were counted daily for thirty days, using a stereomicroscope, applying the botanical criterion for germination, which includes the emergence of the embryo from the seed coat and the curving of the radicle [27]. To statistically quantify germination, the following indices were used: Germination percentage (G %), which determines the percentage of germinated seeds relative to the total number of seeds placed in the experiment: G % = (Ng × 100)/Nt, where Ng is the number of germinated seeds, and Nt is the total number of seeds [17]. The germination speed index (GSI) is calculated as GSI = G1/N1 + G2/N2 + … + Gn/Nn, where Gn is the number of germinated seeds and Nn is the number of days of germination [27]. The Synchrony Index, given by the formula E = −∑fi. log_2_fi refers to the synchronization of germinations under different conditions [30]. These indices were compared using two-way ANOVA and Tukey’s test at 5% in Rstudio [40]. Seed and seedling features were photographed with the help of a digital camera. Measurements were made with a digital caliper.

## 5. Conclusions

We hypothesized that the species is protandrous, a hypothesis that was confirmed in our study. It was observed that, during the anthesis period, which can last up to nine days, the flowers exhibit functional differentiation, initially acting as pollen donors and later as pollen receivers. In contrast to most species of the genus that have been previously studied, *A. integerrima* is self-compatible, which facilitates fruit production through both allogamy and pollinator-promoted geitonogamy [5,6]. The primary pollinators are oil-collecting bees, which are highly efficient and contribute to the species achieving its maximum fruiting potential, as the flowers depend on these agents for fruiting. Optimal germination was consistent across all populations, as expected, with 25 °C being the ideal temperature, resulting in nearly 50% seed germination. The populations exhibited different vigor levels, with the larger population producing more vigorous seeds. Germination is an effective, economical, and rapid propagation method for *A. integerrima*, due to several factors: (1) nearly 50% of the seeds germinate at the ideal temperature; (2) germination begins by the third day; (3) the seeds are non-dormant, making the process faster and more economical; and (4) all plants that germinated in this experiment flowered within one year of cultivation (personal observations). The rapid transformation of habitats has posed challenges to the survival of native species; however, it is now known that urban environments host a significant diversity of native nesting bees [41]. Urban gardens play a crucial role as refuges for these species, with the presence of flowers offering food resources essential for their survival. In urban areas where native species are cultivated, the abundance of native bees tends to be significantly higher [12]. The main pollinators of *A. integerrima* are also known to play an important role as pollinators of local Orchidaceae, Malpighiaceae and Gesneriaceae [42,43,44]. Furthermore, Plantaginaceae includes species with a high production of secondary metabolites of pharmacological interest, such as *Digitalis purpurea* L., which is widely recognized for its diverse phytochemical compounds with medicinal potential, although highly toxic [45]. Thus, we emphasize the importance and necessity of phytochemical and pharmacological approaches to investigate the medicinal potential and toxicity of *A. integerrima*. Finally, the ornamental cultivation of *A. integerrima* represents an effective strategy for promoting native flora conservation, reducing the risk of invasion by exotic species, and increasing the availability of resources for native pollinators.

## Figures and Tables

**Table 1:** Number of interactions of bee species, foraged resource, and time of visits (seconds) on *Angelonia integerrima* Spreng. flowers.

Species	Total Interactions	Foraged Resource	Total Duration of Interactions (s)	Average Duration of Interactions (s)	Standard Deviation
*Centris trigonoides*	59	oils	203	2.44	±1.77
*Centris* sp.	48	oils	84	1.75	±1.45
*Tetrapedia diversipes*	10	oils/pollen	69	6.9	±7.17
*Dialictus* sp.	13	pollen	725	55.76	±41.49

**Table 2:** *Angelonia integerrima* fruiting in self-pollination, cross-pollination, emasculation, and control crosses. %: number of fruits/number of flowers; N: number of plants used in the experiments. ANOVA *p*-value for self-pollination and cross-pollination treatments.

Species	N	Self-Pollination	Cross-Pollination	Emasculation	Control	*p*-Value
*Angelonia integerrima*	17	48.6% (18/37)	62% (23/37)	0% (0/45)	0% (0/32)	0.0573

**Table 3:** Germination percentage (G %), germination speed index (GSI), and synchrony (E) values in different populations: Jardim Botânico (JB), Morro Santana (MS), and Morro São Pedro (MSP). Values with the same letters within each row did not differ statistically according to Tukey’s test at 5%. The values of each line are minimum–maximum (mean).

	Jardim Botânico (JB)	Morro Santana (MS)	Morro São Pedro (MSP)
G (%)	0–48 (22.93) a	8–64 (26.25) a	16–64 (41.75) b
GSI	0–4.75 (2.08) a	0.30–9.39 (270) ab	1.80–7.40 (4.08) b
E	0–2.85 (1.63) a	0–285 (1.80) a	0–2.83 (1.98) a

**Table 4:** Germination percentage (G %), germination speed index (GSI), and Synchrony (E) values at different temperatures: control (29.2 °C), 20 °C, 25 °C, and 30 °C. Values with the same letters within each row did not differ statistically according to Tukey’s test at 5%. The values of each line are minimum–maximum (mean).

	Control	20 °C	25 °C	30 °C
G (%)	4–24 (17) a	8–64 (27.66) a	28–64 (49.33) b	0–60 (27.25) a
GSI	0.5–3.63 (2.03) a	0.69–6.28 (2.35) a	2.18–9.39 (5.19) b	0–5.66 (2.26) a

## Data Availability

Data are available upon request from the correspondent author.

## Supplementary Materials

The following supporting information can be downloaded at https://www.mdpi.com/article/10.3390/plants14050663/s1: Video S1: Pollinators of *Angelonia integerrima*.
